# Estimating the effect of mobility on SARS-CoV-2 transmission during the first and second wave of the COVID-19 epidemic, Switzerland, March to December 2020

**DOI:** 10.2807/1560-7917.ES.2022.27.10.2100374

**Published:** 2022-03-10

**Authors:** Adrian Lison, Joel Persson, Nicolas Banholzer, Stefan Feuerriegel

**Affiliations:** 1 ETH Zurich, Zurich, Switzerland; 2 LMU Munich, Munich, Germany

**Keywords:** COVID-19, SARS-CoV-2, Coronavirus, transmission, effective reproduction number, mobility, mobile phone, Switzerland

## Abstract

**Introduction:**

Human mobility was considerably reduced during the COVID-19 pandemic. To support disease surveillance, it is important to understand the effect of mobility on transmission.

**Aim:**

We compared the role of mobility during the first and second COVID-19 wave in Switzerland by studying the link between daily travel distances and the effective reproduction number (*R_t_*) of SARS-CoV-2.

**Methods:**

We used aggregated mobile phone data from a representative panel survey of the Swiss population to measure human mobility. We estimated the effects of reductions in daily travel distance on *R_t_* via a regression model. We compared mobility effects between the first (2 March–7 April 2020) and second wave (1 October–10 December 2020).

**Results:**

Daily travel distances decreased by 73% in the first and by 44% in the second wave (relative to February 2020). For a 1% reduction in average daily travel distance, *R_t_* was estimated to decline by 0.73% (95% credible interval (CrI): 0.34–1.03) in the first wave and by 1.04% (95% CrI: 0.66–1.42) in the second wave. The estimated mobility effects were similar in both waves for all modes of transport, travel purposes and sociodemographic subgroups but differed for movement radius.

**Conclusion:**

Mobility was associated with SARS-CoV-2 *R_t_* during the first two epidemic waves in Switzerland. The relative effect of mobility was similar in both waves, but smaller mobility reductions in the second wave corresponded to smaller overall reductions in *R_t_*. Mobility data from mobile phones have a continued potential to support real-time surveillance of COVID-19.

## Introduction

The coronavirus disease (COVID-19) pandemic had repeated surges of community transmission in many European countries [1]. In Switzerland, the effective reproduction number (*R_t_*) of severe acute respiratory syndrome coronavirus 2 (SARS-CoV-2) surpassed the threshold of 1 during both a first wave in spring 2020 and a second wave in autumn 2020. To enable timely surveillance of the epidemic, the use of mobile phone data has been proposed [2-4]. Mobile phone data can capture human movements in near-real time and thus serve as a proxy for population-level mobility under COVID-19 policy measures [5-7]. Using such mobility data, early studies have shown that human migration out of Wuhan, China, played a pivotal role in the initial spreading of COVID-19 in China [8,9]. Moreover, mobility data can be linked to epidemiological indicators such as growth rates or *R_t_* to analyse the relationship between mobility and disease transmission [10,11]. Insights into this relationship provide an opportunity for real-time monitoring of epidemic trends [3,4] and have implications for the effectiveness of policy measures aimed at reducing mobility [12,13].

For the first wave of the COVID-19 epidemic, mobility was consistently associated with infections [14-18], deaths [19], confirmed cases [20,21], case growth rates [10,22] and *R_t_* [11,23-25]. This suggests an important role of mobility reduction for epidemic control during the first wave. Studies analysing longer time periods, however, suspected that the relationship between mobility and disease transmission may have weakened over time. For example, Badr et al. found a strong correlation between the number of daily trips and the case growth rate for the first wave in the United States [10], but not for later time periods [26,27]. Similarly, Nouvellet et al. estimated a dampened, non-significant or even reversed relationship between mobility and *R_t_* after May 2020 for 42 of 52 countries analysed [28]. To date, few studies have included data from later waves to analyse the relationship between mobility and transmission [25,29]. It is thus important to understand whether mobility reduction continued to be an important means of epidemic control in later waves, despite increased hygiene measures, contact tracing and population awareness.

In this study, our aim was to compare the role of mobility during the first and the second epidemic wave of COVID-19 in Switzerland. Our analysis focused on epidemic waves, because we expect population-level behaviour to be most relevant for disease control under large-scale community transmission. For each wave, we estimated the effect of human mobility, measured by daily travel distances, on *R_t_* of SARS-CoV-2. We compared the estimated mobility effects between the first and second wave. We further analysed how the estimated effects varied when using mobility data of only a certain mode of transport, travel purpose or sociodemographic subgroup (age group and employment status). We also estimated how the movement radius was linked to *R_t_*.

## Methods

### Study periods

The study periods were selected based on criteria by the World Health Organization to indicate whether an epidemic is under control [30]. Specifically, the selected study periods had both a high average number of transmissions per infectious person (indicated by *R_t_* > 1) and a substantial proportion of infectious persons (indicated by a test positivity rate above 5%) in the previous 3 weeks. For Switzerland, this resulted in two study periods from 2 March to 7 April 2020 and from 1 October to 10 December 2020, hereafter referred to as first and second wave. The end of the second study period was set to 2 weeks before Christmas Eve to exclude changes in reporting practice during the Christmas holidays.

### Mobility data

We measured human mobility using data from a mobile phone-based panel survey. The panel survey was conducted by intervista AG (Bern, Switzerland) on behalf of the Swiss National COVID-19 Science Task Force. It included 2,561 participants that were representative of the population in Switzerland by age, sex and region [31]. Moreover, the age groups (15–29, 30–64 or 65–79 years) and employment status (employed, unemployed or in education) of each participant were recorded. Details on the representativeness and stability of the panel are provided in the Supplement, section A. Daily releases of the data were made publicly available throughout the epidemic.

Movements of each participant were continuously tracked throughout the year 2020 via triangulation between cell towers and Wi-Fi hotspots, data from movement sensors of the mobile phone and interactions with Bluetooth beacons [31]. Based on the movement data, the absolute daily travel distance and the movement radius (both in km) were computed for each participant and aggregated over the study population. Analogous to the estimates of *R_t_*, the aggregates were smoothed with a 3-day moving average. The movement radius is defined as the maximum distance from the place of residence on a given day. Moreover, statistical models using movement data, information on sites and participant profiles were employed to infer the mode of transport (car/motorcycle, public transport, foot or other) and travel purpose (occupation, shopping or leisure) of each trip. Details on the definition of the categories are provided in the Supplement, section A.

### Effective reproduction number and policy measures

*R_t_* denotes the expected number of secondary infections resulting from an infection at time *t* and is used to monitor disease transmission over time. We obtained estimates of *R_t_* in Switzerland from the Swiss National COVID-19 Science Task Force [32,33]. The underlying estimation procedure was based on the time series of newly hospitalised patients with COVID-19 and adjusted for the incubation period and time between symptom onset and hospitalisation. Because of this adjustment, *R_t_* attributes transmission precisely on day *t* without any time lag. The estimates were provided as a 3-day moving average. We used point estimates of *R_t_* for our analysis. In the descriptive plots, we also report uncertainty intervals.

We selected relevant COVID-19 policy measures in Switzerland using a systematic procedure. We obtained implementation dates of the policy measures from the official regulations of the Swiss Federal Council and checked them against dates from the Oxford Government Response Tracker [34] and the Swiss National COVID-19 Science Task Force [32]. Details on the systematic procedure and encoding of policy measures are provided in the Supplement, section A. For the first wave, the dates of policy measures were encoded as 13 March 2020 (ban on public gatherings of more than 100 people), 17 March 2020 (closures of schools and venues) and 21 March 2020 (ban on public gatherings of more than five people). For the second wave, the dates of policy measures were encoded as 19 October 2020 (ban on public gatherings of more than 15 people) and 28 October 2020 (venue restrictions and ban on private meetings of more than 10 people). Each of these measures were implemented nationwide (i.e. across all cantons in Switzerland).

### Statistical analysis

We linked mobility to *R_t_* of SARS-CoV-2 using a regression model. As in earlier research [35], we assumed that *R_t_* on day *t* followed a gamma distribution, thus also accounting for the fact that *R_t_* cannot be negative. We then modelled a log-linear relationship between the expected value of *R_t_* and the observed average daily travel distance on the same day. We further applied a logarithmic transformation such that changes in mobility could be measured on a relative scale. We accounted for the effects of policy measures by adding dummy variables indicating whether a measure was in effect on day *t* or not. This was done because policy measures may reduce *R_t_* not only through changes in mobility but also by other means of transmission reduction (e.g. physical distancing in public, prevention of large gatherings or hygiene measures and face mask use) and are thus potential confounders of the relationship between mobility and *R_t_*. For each weekday, a different intercept was included to capture potential differences between weekdays in reporting and mobility (e.g. owing to less reporting and mobility on weekends). In the resulting model, the coefficient of mobility can be interpreted as the expected percentage change in *R_t_* associated with a 1% change in mobility, conditional on the policy measures and the day of the week.

We further wanted to assess the sensitivity of the estimated mobility effect to the use of mobility data from a particular subpopulation or of a certain type of mobility. Therefore, we fitted a separate model for each mode of transport, for each travel purpose and for each sociodemographic subgroup, with the stratified average daily travel distance as explanatory variable. All other variables were analogous to the main model from above.

We also analysed the extent to which the movement radius was linked to *R_t_*. The movement radius was categorised into residential (*<* 500 m), local (500 m–2 km), municipal (2–10 km), regional (10– 50 km) or long-range (*>* 50 km) mobility. The daily share of the study population travelling within each movement radius was computed and, for each movement radius, a separate regression model was fitted where that share was the explanatory variable. Here, the coefficient of movement radius can be interpreted as the expected percentage change in *R_t_* associated with an increase of one percentage point in the share of mobility within a certain movement radius. Otherwise, the model had the same specification as the model based on travel distance.

The specifications of the different models are provided in the Supplement, section B. The models were fitted separately for the first wave (using data from 2 March to 7 April 2020) and the second wave (using data from 1 October to 10 December 2020). This enabled us to estimate the effects of mobility independently for both waves, while respecting potential differences in epidemiological features or reporting practices.

All model parameters were estimated in a fully Bayesian framework. Estimation was conducted in R 4.0.3 and Stan 2.01.0 using Markov chain Monte Carlo sampling via the No-U-Turn sampler. Four chains were run, with 1,000 warm-up iterations and 1,000 sampling iterations each. We defined weakly informative priors for all model parameters (see Supplement, section B), giving conservative estimates for the effect of mobility through appropriate regularisation. The estimates were checked using common Bayesian model diagnostics and indicated good model fit, sufficient effective sample size, convergence of the chains and absence of particularly influential observations (see Supplement, section C). Unless stated otherwise, we report the posterior mean and the 95% credible interval (CrI) of estimated parameters, which can be interpreted as containing the quantity of interest with high probability.

As part of our robustness checks, we tested alternative estimates of *R_t_*, other study periods and stratification by further sociodemographic subgroups. Moreover, we extended our model with autocorrelated error terms and accounted for potential changes in testing intensity. For comparison, we also fitted models not accounting for policy measures. All checks confirmed the robustness of our results.

### Ethical statement

Movement data from the panel survey were collected in anonymised form in line with the Federal Act on Data Protection and the General Data Protection Regulation. All participants consented to the use for scientific purposes. Only aggregated data were analysed in this study. Ethics approval for the study was obtained by the institutional review board at ETH Zurich (reference number EK 2020-N-179).

## Results

**Figure 1:**
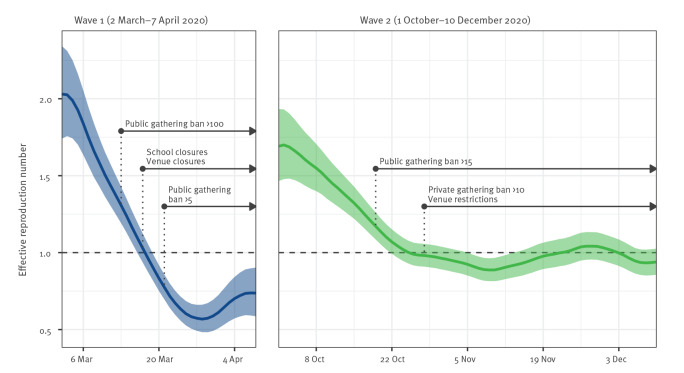
Effective SARS-CoV-2 reproduction number during the first and second COVID-19 wave, Switzerland, 2 March–7 April and 1 October–10 December 2020

COVID-19: coronavirus disease; SARS-CoV-2: severe acute respiratory syndrome coronavirus 2.

*R_t_* is shown in Figure 1 for both study periods: 2 March to 7 April 2020, i.e. the COVID-19 first wave, and 1 October to 10 December 2020, i.e. the second wave, in Switzerland. During both waves, *R_t_* was initially above the threshold of 1 (indicating exponential growth) but decreased over time. In the first wave, *R_t_* fell below the threshold of 1 from 18 March 2020 onwards. In the second wave, *R_t_* first fell temporarily below the threshold of 1 on 26 October 2020 but surpassed the threshold again on 23 November 2020.

Shaded areas: 95% credible interval. Annotations show the implementation dates of policy measures. Once introduced, policy measures remained in effect until the end of both study periods.

### Changes in mobility

**Figure 2:**
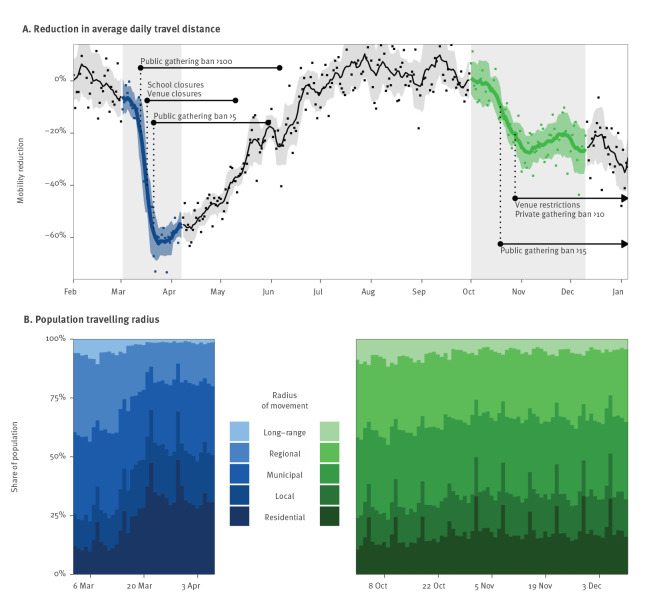
Human mobility during the first and second COVID-19 wave, Switzerland, 2 March–7 April and 1 October–10 December 2020 (n = 2,561)

We measured relative reductions in mobility during the epidemic as percentage change from the average daily travel distance in February 2020. As shown in Figure 2A, mobility decreased considerably in both waves. In the first wave, a large reduction in daily travel distances occurred from 13 March 2020 onwards. The strongest change was reached on 29 March 2020, with a reduction of 73.37%. In the time period between the first and second wave, mobility increased, and, as a result, daily travel distances were of a similar magnitude during the period from July to October as in February. In the second wave, the reduction in daily travel distances was more gradual and of smaller size. Here, the strongest change was reached on 6 December 2020 with a reduction of 43.63%.

COVID-19: coronavirus disease.

Panel A: reductions in average daily travel distance relative to the February average. Dots: daily reductions, shaded trend lines: corresponding 7-day moving average with standard deviation. Annotations show the implementation dates of policy measures. Panel B: share of the population travelling within a certain movement radius. Weekly peaks in the residential and local radius highlight decreased mobility on Sundays.

The share of mobility within a smaller movement radius increased considerably in the first wave and slightly in the second wave (Figure 2B). For example, the share of the population travelling within a residential radius (*<* 500 m) was at most 28.50% in February, whereas it reached up to 48.29% (19 March 2020) in the first wave and up to 33.00% (29 November 2020) in the second wave. In contrast, the share of the population travelling within a long-range radius (*>* 50 km) decreased. The share accounted for up to 6.03% of the study population in February, but the share declined to 1.15% (31 March 2020) in the first wave and 3.59% (4 November 2020) in the second wave.

**Figure 3:**
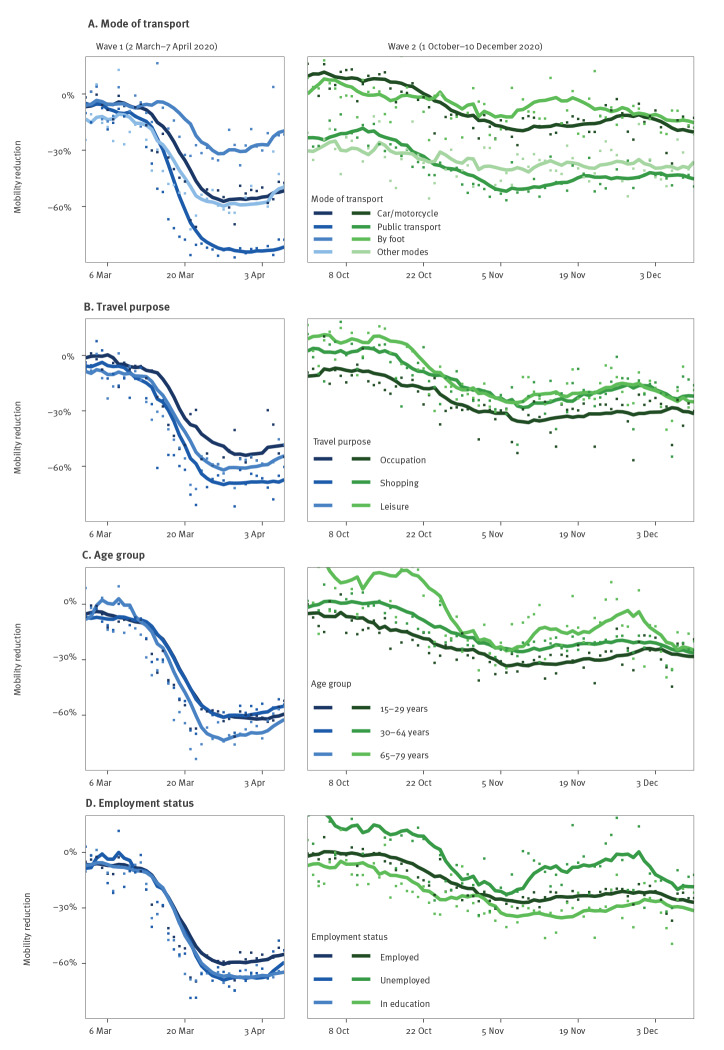
Comparison of relative mobility reductions over time during the first and second COVID-19 wave, Switzerland, 2 March–7 April and 1 October–10 December 2020 (n = 2,561)

Relative mobility reductions varied notably across modes of transport, with walking affected least and public transport affected most (Figure 3A). As such, daily travel distances by public transport reached a maximum reduction of 87.23% (29 March 2020) in the first wave and 56.72% (6 November 2020) in the second wave. They were also lower than the average in February 2020 during the time between the waves. Daily travel distances by car/motorcycle decreased considerably in the first wave with a maximum reduction of 70.94% (22 March 2020), but less strongly in the second wave with a maximum reduction of 37.09% (6 December 2020). Across different travel purposes, mobility reductions were mostly similar in both waves (Figure 3B). Across different age groups and employment statuses, mobility also showed broadly similar declines (Figure 3C-F).

COVID-19: coronavirus disease.

For both waves, reductions in average daily travel distance, relative to the February average, are shown. Dots: daily reductions, trend lines: corresponding 7-day moving average.

### Estimated effects of mobility

**Figure 4:**
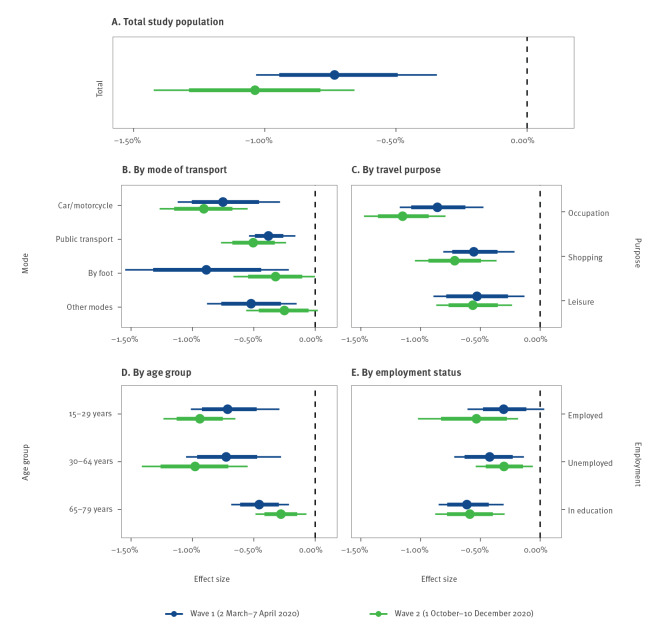
Estimated relative effects of mobility on the effective reproduction number of SARS-CoV-2, Switzerland, 2 March–7 April and 1 October–10 December 2020 (n = 2,561)

We estimated the effect of mobility on *R_t_* separately for both waves using our regression model. The estimated coefficients for the effect of mobility were of similar magnitude in the first and second wave. As shown in Figure 4A, the estimated reduction in *R_t_* per 1% reduction in average travel distance of the population was 0.73% (95% CrI: 0.34–1.03) in the first wave and 1.04% (95% CrI: 0.66–1.42) in the second wave. The relative effect of mobility between the first and second wave was similar when analysing mobility data from all modes of transport, travel purposes, age groups and employment statuses. In all cases, the 80% CrI for mobility effects overlapped between the first and second wave (Figure 4B-G). Further sociodemographic subgroups were analysed as part of our robustness checks and showed similar results (see the Supplement, section D for those additional comparisons).

SARS-CoV-2: severe acute respiratory syndrome coronavirus 2.

Shown are the estimated changes in the effective reproduction number given a 1% reduction in mobility, for the first and second wave, respectively. Posterior means (dots) and the 80% and 95% credible intervals (thick and thin bars) are reported.

**Figure 5:**
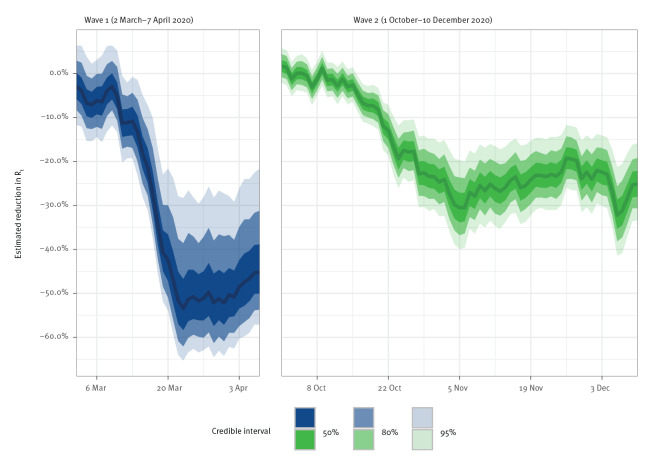
Estimated overall effect of mobility on the effective reproduction number of SARS-CoV-2, Switzerland, 2 March–7 April and 1 October–10 December 2020 (n = 2,561)

Figure 5 shows the estimated overall effect of observed mobility reductions during the first and second wave. As the observed reduction in mobility was larger for the first wave than the second wave, the overall change in *R_t_* was also larger for the first wave (even though the estimated relative mobility effects were similar). In the first wave, the maximum overall reduction in *R_t_* attributed to mobility by the model was estimated to be 51.56% (95% CrI: 28.30–65.28). Together with the implemented policy measures, this corresponds to an estimated *R_t_* of 0.61 (95% CrI: 0.47–0.78), which is below the threshold of 1 with high probability. In the second wave, the maximum overall reduction was estimated to be 31.94% (95% CrI: 21.27–41.44). Together with the implemented policy measures, this corresponds to an *R_t_* of 0.87 (95% CrI: 0.75–1.00), which is not below the threshold of 1 with high probability. Hence, the comparatively smaller mobility reductions in the second wave also resulted in smaller reductions of *R_t_*.

*R_t_*: effective reproduction number; SARS-CoV-2: severe acute respiratory syndrome coronavirus 2.

Shown are the daily estimated reductions in the effective reproduction number attributed to mobility by the model in the first and second wave. Posterior means (lines) and 50%, 80% and 95% credible intervals (shaded areas) of *R_t_* reductions are reported.

**Figure 6:**
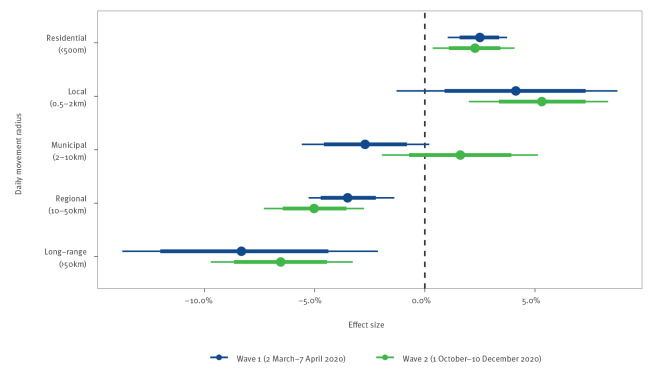
Estimated mobility effects on the effective reproduction number of SARS-CoV-2 across movement radius, Switzerland, 2 March–7 April and 1 October–10 December 2020 (n = 2,561)

We furthermore analysed the extent to which the movement radius was linked to *R_t_* (Figure 6). The estimated effects of a residential, local, regional and long-range movement radius were similar in both waves, with the corresponding 80% CrI for the two waves overlapping. *R_t_* was negatively associated with the residential movement radius and positively associated with both the regional and the long-range movement radius in both waves. The local movement radius had a significant association with *R_t_* only in the second wave, when the estimated change in *R_t_* per one percentage point reduction in the population share was 5.31% (95% CrI: 2.00–8.32). Moreover, the effect of the municipal movement radius probably differed between the two waves. In the first wave, we estimated a −2.71% (95% CrI: −5.59 to 0.20) change in *R_t_* per one percentage point reduction in the corresponding population share. In the second wave, the same effect was estimated to be 1.61% (95% CrI: −1.94 to 5.14).

*R_t_*: effective reproduction number; SARS-CoV-2: severe acute respiratory syndrome coronavirus 2.

Shown are the estimated changes in *R_t_* given a one percentage point decrease in the share of the population travelling within a certain movement radius. Posterior means (dots) and the 80% and 95% credible intervals (thick and thin bars) are reported.

### Robustness checks

To assess the robustness of our results, we tested other study periods for defining the first and second wave, other estimates of *R_t_*, an extended model with autocorrelated error terms and a model adjusting for the effect of testing intensity (see Supplement section D for the detailed analyses). In all tests, the estimated effects of mobility for both waves were robust. The estimated effects from models not accounting for policy measures were slightly larger, confirming the presumed confounding of policy measures and thus motivating the inclusion as control variables. In addition, we found no significant effect of mobility when fitting our model for the time period in between the first and the second wave, which generally corresponded to a phase with fairly low case numbers.

## Discussion

In this work, we studied how mobility was linked to *R_t_* of SARS-CoV-2 in Switzerland. For this, we obtained mobility data from a mobile phone-based panel survey whose participants were representative of the population by age, sex and region. Our research on the link between mobility and COVID-19 spread extends beyond previous studies by comparing the effect of mobility in two different epidemic waves [10,11,14-25].

As our results show, mobility in Switzerland declined considerably during the first wave (defined as 2 March to 7 April 2020) and to a lesser extent in the second wave (1 October to 10 December 2020). This observation can reflect differences in population behaviour and the policies adopted by the Swiss government. In particular, while venue closures led to an overall shutdown in the first wave, policies in the second wave initially focused on contact tracing and hygiene measures with fewer restrictions on public life [36], a pattern which has been observed in many European countries [34]. Furthermore, the relative reductions in mobility were similar across travel purposes and sociodemographic subgroups, implying mostly homogeneous mobility changes among the population in Switzerland. We observed differences in mobility reduction between modes of transport, indicating a tendency of the population to avoid public transport.

Using our regression model, we linked reductions in mobility to changes in *R_t_* of SARS-CoV-2 for the first and second COVID-19 wave in Switzerland. Our results for the first wave are consistent with previous studies on that wave, which have found a positive association between mobility and transmission [10,11,14-25]. For Switzerland, Lemaitre et al. measured a strong correlation between changes in *R_t_* and changes in the number of visits and lengths of stay at work places, transit stations, retail and recreation sites and residential areas [24]. Some studies have also estimated mobility effects for the first wave. For example, Unwin et al. have estimated an *R_t_* reduction of 37% (95% CrI: 16–56) for an observed mobility reduction of 62% in the United States [11]. Using data from 11 European countries, Bryant et al. estimated a median *R_t_* reduction of between 0.03% and 3.08% for a one percentage point reduction in mobility, depending on the type of mobility [23]. These results overlap with our estimates for the first wave in Switzerland. Importantly, the above studies all relied on data from Google mobility reports which measure visits at specific points of interest. A notable finding of our work is that similar results are obtained when using representative mobility data that capture individual movements.

For the second wave, we found that the effect of a 1% reduction in average daily travel distance on *R_t_* was of similar magnitude as in the first wave. Our results therefore indicate that population-level measures of mobility serve as a meaningful proxy for population mixing and contact rates that can explain changes in transmission during different epidemic waves. Previous work has suspected that, for time periods after the first wave, the relationship between mobility and transmission could have weakened in many countries [26-28]. This change has been attributed to potential confounding by policy measures and individual behaviour, questioning the continued value of policy measures targeted at human mobility reductions. For further policymaking, it should therefore be assessed whether the role of mobility was diminished in the later course of the epidemic, for example because of increased hygiene measures, contact tracing and population awareness. The results of our study suggest that human mobility remained an important determinant for explaining reductions in SARS-CoV-2 transmission also during the second wave in Switzerland.

Our findings were consistent across mobility data for different modes of transport, travel purposes and sociodemographic subgroups. We have furthermore confirmed the robustness of our estimates against alternative model specifications using extensive robustness checks. Importantly, it may well be that the knowledge of policymakers and the public about *R_t_* on a given day influences mobility behaviour on later days. However, it cannot influence mobility on the same day, as information about new infections and *R_t_* only becomes available with a substantial delay. Our estimated mobility effects can therefore not be biased by a reverse effect of *R_t_* on mobility.

The two waves differed with regard to the absolute effect of mobility, since the smaller mobility reductions observed in the second wave also resulted in smaller absolute reductions of *R_t_*. We further found that, in the first wave, reductions of daily movements beyond a local radius were more consistently associated with reductions in *R_t_*, suggesting a more pronounced role of stay-at-home behaviour during the first wave. This is likely to reflect the overall shutdown of public life, where contacts were reduced by restricting daily movements to a necessary minimum.

Our results have several implications for policymaking. Firstly, a significant association between human mobility and transmission during epidemic waves indicates that mobility data allow monitoring of physical distancing behaviour. Secondly, the similar relative effects in both waves imply that mobility reductions can be effective in limiting transmission also in later waves. Finally, mobility reductions in the second wave were smaller than in the first wave, suggesting that further transmission reductions could have been achieved with more physical distancing.

This study is subject to several limitations. Firstly, our analysis was limited to Switzerland. While the overall course of the epidemic in Switzerland was characterised by a similar variability as in many European countries, a future comparison with other countries to investigate country-specific differences in mobility behaviour could be especially valuable. Secondly, we used mobility data only from a sample of the population in Switzerland, which was however selected to be representative by age, sex and region. Thirdly, in our study, mobility was measured via the daily travel distance. Other studies have analysed mobility in the first wave using alternative metrics such as the radius of gyration or trip counts [2], which may have different interpretations. However, as our model measures changes in mobility on a relative scale, we expect that our results are not highly sensitive to the choice of metric. Fourthly, mode of transport and travel purpose were indirectly inferred from movement data using statistical models. These may be subject to errors but were validated against mobility census data by the data provider. Fifthly, in our models, both the mobility variables and the time-varying *R_t_* were smoothed with a 3-day moving average. Our estimates are therefore with respect to smoothed mobility and *R_t_*. We further acknowledge that the estimation of *R_t_* can entail considerable uncertainty, especially when the number of reported cases is small. We addressed this by selecting only study periods with a substantial proportion of infectious persons and thus comparatively small uncertainty. Sixthly, to capture structural changes in transmission aside from mobility, we fitted separate models for both the first and second wave and included variables for policy measures and weekday-specific intercepts. Still, it cannot be ruled out that there were further factors not included in our model that could explain the changes in mobility and *R_t_*. Our estimates should therefore be interpreted as providing associative and not causal relationships. Finally, it is important to note that our study estimated the overall effect of mobility on *R_t_*. We thereby did not model the effects of other behavioural changes such as mask wearing, which could have moderated the effect of mobility.

## Conclusion

Our study highlights the continued value of mobile phone data in the context of real-time disease surveillance of COVID-19. For both the first and second wave in Switzerland, we provide evidence that changes in the time-varying *R_t_*, which becomes available only with a time lag of several days or weeks, can be predicted by mobility data available on the same day. Thus, digital tracking of human movements may provide an opportunity for real-time assessment of the epidemic situation, ahead of traditional reporting. Our findings further suggest that mobility reduction continues to be a relevant factor in epidemic control, despite increased hygiene measures, contact tracing, and population awareness as present in the second wave. Policy measures aimed at limiting population mixing and contact rates through mobility reduction is likely to remain an instrument in the COVID-19 public health response to manage further epidemic waves until population immunity is achieved. Here, a challenge for policymakers will be to balance mobility and its effects on transmission in the context of increasing vaccination rates and spreading SARS-CoV-2 variants of concern. In the light of this challenge, mobility data from mobile phones can contribute to a timely and informed public health response.

## Supplementary Data

517000 Supplement
